# Academic performance and use of psychoactive drugs among healthcare students at a university in southern Brazil: cross-sectional study

**DOI:** 10.1590/1516-3180.2019.0182.R1.21102019

**Published:** 2020-04-22

**Authors:** Karine de Lima Sírio Boclin, Fernanda Fabian Callejon Cecílio, Gabriela Faé, Gabriela Fanti, Guilherme Centenaro, Thoany Pellizzari, Emanuela Gaviolli, Débora Nunes Mario, Lilian Rigo

**Affiliations:** I PhD. Professor, School of Medicine, Universidade Estácio de Sá (UESA), Rio de Janeiro (RJ), Brazil.; II Undergraduate Student, School of Medicine, Faculdade Meridional (IMED), Passo Fundo (RS), Brazil.; III Undergraduate Student, School of Medicine, Faculdade Meridional (IMED), Passo Fundo (RS), Brazil.; IV Undergraduate Student, School of Medicine, Faculdade Meridional (IMED), Passo Fundo (RS), Brazil.; V Undergraduate Student, School of Medicine, Faculdade Meridional (IMED), Passo Fundo (RS), Brazil.; VI Undergraduate Student, School of Medicine, Faculdade Meridional (IMED), Passo Fundo (RS), Brazil.; VII MSc. Dental Surgeon, at a private clinic, Passo Fundo (RS), Brazil.; VIII PhD. Professor, School of Medicine, Universidade Federal do Pampa (UNIPAMPA), Uruguaiana (RS), Brazil.; IX PhD. Professor, School of Dentistry, Postgraduate Program on Dentistry, Faculdade Meridional (IMED), Passo Fundo (RS), Brazil.

**Keywords:** Students, medical, Students, dental, Drug effects [Subheading], Psychoactive drugs, Academic performance, Illicit drugs, Licit drugs, Higher education institution

## Abstract

**BACKGROUND:**

People have been using psychoactive substances for a long time. Over the last few years, this practice has spread among university students, who use these substances to improve their academic performance, relieve stress and increase concentration and memory.

**OBJECTIVES:**

To estimate the use of psychoactive drugs among healthcare students at a higher education institution in the city of Passo Fundo (RS), Brazil, and to ascertain the associated demographic and lifestyle factors.

**DESIGN AND SETTING:**

Cross-sectional study in a higher education institution.

**METHODS:**

We included 287 undergraduate medicine and dentistry students in this study. They answered a self-administered questionnaire regarding sociodemographic, lifestyle and health variables. The statistical analysis used univariate and bivariate analyses with Pearson’s chi-square test (P-value < 0.05). ­Multivariate analyses were used to estimate odds ratios (OR) and their respective 95% confidence intervals. The SPSS software, version 20.0, was used.

**RESULTS:**

The prevalence of use of psychoactive substances among the students was 24.7%. Among these students, high frequencies of psychoactive drugs had been prescribed by physicians (95.8%) and for the purpose of relaxation or stress relief (73.2%). Women, medical students (compared with dental students) and participants with lower academic performance were more likely to use psychoactive drugs. After the multivariate adjustment, the “course” and “academic performance” remained associated with use of psychoactive drugs.

**CONCLUSION:**

There was high prevalence of psychoactive drug use among the students at the higher education institution investigated. Some variables (female sex, medical students and low academic performance) were associated with the outcome.

## INTRODUCTION

Throughout history, humans have resorted to the use of psychoactive substances for various purposes. Psychoactive drugs work on the brain, modifying its operation and changing mood, behavior and consciousness, which may lead to a state of dependence. Among these substances are licit drugs (alcohol and tobacco), illicit drugs (marijuana, cocaine, crack and others) and psychoactive drugs (tranquilizers, sedatives and strong analgesics).^1^

Generations of students have used brain stimulants, food supplements, drugs subject to medical prescription, amphetamines and energy drinks in order to enhance their mental faculties, namely memory, concentration, reasoning and language, which are inherent to studying and academic development.^2^^,^^3^^,^^4^

The main substances used for this purpose are caffeine, methylphenidate (MPH), modafinil, piracetam, energy drinks and amphetamines. Although the specific action mechanisms may vary, psychostimulants usually work directly or indirectly through dopamine^5^^,^^6^, motivation, attention and excitement.^7^

MPH (also known as Ritalin™) and dextroamphetamine (d-AMP) are the most commonly used non-medical substances, although other amphetamine formulations have also been used. However, these substances, which belong to the class of amphetamines, present significant risk of dependence.^8^ Among students using methylphenidate, most of them have used it in highly stressful periods of academic studies.^9^ Thus, studies have estimated that 5% to 35% of university students use these drugs to improve their academic performance, stimulate cognitive function and decrease physical and mental fatigue.^2^^,^^10^

Hence, the increasing use of psychoactive drugs for non-therapeutic purposes creates the need to inform students and raise their awareness about the real risks of over-the-counter psychoactive drugs and their harmful effects on health, such as increased blood pressure, arrhythmias, headaches, overdose and depression.^2^^,^^10^^,^^11^ Therefore, studies providing data on the use of psychoactive substances among healthcare students become relevant. Understanding and reflecting on the issues raised by this topic may allow the creation of effective intervention strategies.

## OBJECTIVE

The aim of the present study was to estimate the use of psychoactive drugs among healthcare students at a higher education institution located in the city of Passo Fundo (RS), Brazil, and to ascertain the associated demographic and lifestyle factors.

## METHODS

### Ethical considerations

This study was approved by the institution’s ethics committee, under investigation no. 2.014.448 and CAAE 66373317.6.0000.5319 (April 12, 2017). The voluntary nature of the participation and the confidential nature of the information were explained to the students. All participants signed a free and informed consent form and there was no conflict of interest among the authors.

### Study population

The analyses presented here form part of a larger cross-sectional study on the sociodemographic, lifestyle and health profiles of healthcare students at a higher education institution located in the city of Passo Fundo (RS), Brazil. Every stage of the study was conducted by the students of the scientific methodology course that was offered by the medical school of this institution in the first semester of 2017.

The target population included medicine and dentistry students of a higher education institution. Out of 327 students enrolled in the first semester of 2017 (169 medical students and 158 dental students), 287 participated in this study (148 medical students and 139 dental students), thus resulting in assessment of 87.8% of the total population.

### Data collection

The data were collected through a self-administered questionnaire containing closed questions, which was applied during a class to those of the students present who had agreed to participate in the study. This questionnaire was produced based on questions retrieved from a previous study on a similar population: “Drug Use - adaptation of a questionnaire proposed by the World Health Organization (WHO)”.^12^

### Outcome variable

“Use of psychoactive drugs” was considered to be the outcome. This was evaluated through the question “Do you use psychoactive drugs? (yes or no)”.

Regarding descriptive variables, psychoactive drug users were questioned about medical prescriptions (yes, no); purpose of use (relaxation or stress relief, sleep stimulation, sleep suppression, pain control, increased cognitive ability, increased attention for studying, anxiety or depression); duration of drug use (less than six months, six months to one year, one to two years or three years or more); frequency of drug use (daily, at least once a week, at least once a month or rarely); need to increase the dose (yes or no); and side effects (reduced appetite, euphoria, anxiety, tachycardia, irritability, tremors or headache).

### Covariables

The variables considered were sex (female or male), age (17 to 20 years old or over 20 years old); marital status (married/cohabitating or separated/widowed/single); course (medicine or dentistry); semester (first to eighth); religious practice (yes or no); living situation (alone or with relatives and/or friends); and expected academic performance (better than or equal to expected or below expected).

### Data analysis

In the univariate and bivariate analyses, summary measurements (absolute and relative frequencies) were calculated. To analyze the statistical differences between the categorical variables, Pearson’s chi-square test was used, in which the results with P-values lower than 5% (P-value < 0.05) were considered statistically significant. In the multivariate analysis, crude and adjusted odds ratios (OR) and their respective 95% confidence intervals (CI) were estimated according to the exposure variables. Variables with P-value < 0.20 in the bivariate analyses were included in the multivariate models. The data were analyzed using the Statistical Package for the Social Sciences (SPSS) software, version 20.0.

## RESULTS

The study population consisted predominantly of female university students aged 20 to 36 years. The population was divided into virtually equal proportions for the dental and medical schools. Table 1 presents the characteristics of the students who were and were not using psychoactive drugs.

**Table 1.** Characteristics of the population of healthcare students at a higher education institution who were using psychoactive drugs (n = 71) and were not using psychoactive drugs (n = 216), in the city of Passo Fundo (RS), Brazil, 2017

	Using		Not using	
	n	%	n	%
Sex				
Male	13	18.3	63	29.2
Female	58	81.7	153	70.8
Age category (years)				
17 to 20	25	35.2	91	42.1
21 to 36	46	64.8	125	57.9
Course				
Medicine	49	69.0	99	45.8
Dentistry	22	31.0	117	54.2
Semester				
I to III	38	53.5	95	44.0
IV to VIII	33	46.5	121	56.0
Religion				
Non-practicing	14	19.7	29	13.4
Some religious practice	57	80.3	187	86.6
Living situation				
Alone	35	4.93	101	46.8
With relatives/friends	36	50.7	115	53.2
Marital status				
Married/cohabitating	-	-	13	6.0
Separated/widowed/single	48	100	203	94.0
Expected academic performance				
Higher than or equal to expected	30	16.7	7	13.5
Below expected	40	42.3	133	61.6
Drugs prescribed by physicians	41	57.7	57.7	38.4
Yes	68	95.7	-	-
No	3	4.3	-	-
Purpose of use			-	-
Relaxation or stress relief	52	73.2		
Sleep stimulation	8	11.3	-	-
Sleep suppression	1	1.4	-	-
Pain control	5	7.0	-	-
Increased cognitive ability	3	4.3	-	-
Increased attention for studying	2	2.8	-	-
Anxiety	-	-	-	-
Depression	-	-	-	-
Duration of drug use				
Less than 6 months	18	25.4	-	-
6 months to 1 year	6	8.3	-	-
1 to 3 years	25	35.3	-	-
3 years or more	22	31	-	-
Frequency of drug use				
Daily	51	71.9	-	-
At least once a week	10	14	-	-
At least once a month	7	9.8	-	-
Rarely	3	4.3	-	-
Need to increase the dose				
Yes	35	49.3	-	-
No	36	50.7	-	-
Side effects				
Yes	42	59.2	-	-
No	29	40.8	-	-

Among the students who were using psychoactive drugs, high frequencies of psychoactive drugs had been prescribed by physicians (95.8%) and with the purpose of relaxation or stress relief (73.2%). Most of these students had been using the drugs for one or two years (35.2%) and were taking them daily (71.9%). It was noted that almost half of the students had needed to increase the dose from the initial drug prescription (49.3%). Many students reported having some side effect (59.2%), such as headaches (23.8%) (Table 1).

Table 2 presents the prevalence of psychoactive drug use stratified according to the covariables. Higher prevalence were observed among women, medical students and participants with lower-than-expected academic performance (P-value < 0.05). As anticipated, the same variables were associated with the use of psychoactive drugs in the crude models. Thus, women, medical students (compared with dental students) and participants with lower academic performance were more likely to use psychoactive drugs. After the multivariate adjustments, the variables of “sex”, “course” and “school performance” remained significant.

**Table 2.** Prevalence (%) and crude and adjusted odds ratio (OR) with respective 95% confidence intervals (95% CI) for psychoactive drug use among healthcare students at a higher education institution in the city of Passo Fundo (RS), Brazil, 2017

	Use of psychoactive substances		P-value*	Crude		Adjusted		P-value**
	n	%		OR	95% CI	OR	95% CI	
Sex								
Male	13	18.3	0.048	1.00	--	1.00	--	0.049
Female	50	81.7		1.83	(0.94-3.58)	2.03	(1.00-4.12)	
Age category (years)								
17 to 20	25	35.2	0.18	1.00	--	--	--	0.441
21 to 36	46	64.8		0.74	(0.42-0.30)	--	--	
Course								
Dentistry	22	31.0	0.001	1.00	--	1.00	--	0.010
Medicine	49	69.0		2.63	(1.48-4.65)	2.35	(1.23-4.49)	
Semester								
I to III	38	53.5	0.104	1.00	--	--	--	0.442
IV to VIII	33	43.5		1.46	(0.85-2.51)	--	--	
Religion								
Non-practicing	14	19.7	0.137	1.00	--	--	--	
Some religious practice	57	80.3		1.58	(0.78-3.20)	--	--	
Expected performance								
Higher than or equal to expected	30	42.3	0.003	1.00	--	1.00	--	0.039
Below expected	41	57.7		0.64	(0.26-0.78)	1.872	(1.03-3.23)	

## DISCUSSION

The aim of the present study was to estimate the use of psychoactive drugs among healthcare students at a higher education institution located in the city of Passo Fundo (RS), Brazil, and to ascertain the associated demographic and lifestyle factors. It was observed that most of the university students interviewed who were using psychoactive drugs were obtaining them through medical prescription (95.8%). Among these, the majority reported using this type of medication for relaxation or stress relief (73.2%).

For young people today, university is the time for preparation to face the professional world. This is an important step in an increasingly competitive and challenging society. During the academic course, students feel that they are under pressure to succeed and feel the need to continually surpass themselves. Hence, improvement of concentration, memory and attention is the need that leads university students to misuse psychoactive drugs.^8^

Previous studies targeting student populations have indicated that the prevalence rates for the use of stimulants are between 1% and 38%.^8^ The reasons for their use that university students have stated include optimization of studies, increased concentration, compensation for sleep deprivation and enhancement of moments of relaxation.^8^^,^^13^

According to Wilens et al.,^10^ higher education requires a certain level of development of cognitive function and, in medical schools, this level is particularly high. Thus, students use brain stimulants to increase academic performance.

This study showed an association between the use of psychoactive drugs and women. This result agrees with the findings from other studies such as the one by Lemos et al.,^14^ which showed that there was higher alcohol consumption among men and higher use of tranquilizers among women. Boskovitz et al.^15^ concluded that while men were more likely to consume alcohol, solvents, marijuana and cocaine, women were more likely to use tranquillizers.

According to Simoni-Wastila et al.,^16^ there is a pattern of medicalization among adult women, because they seek relief for their domestic or professional problems through “chemical tranquilizers”.

The present study also found that psychoactive drug use had an influence on school performance. In a previous study, it was observed that a slight increase in concentration and motivation was achieved through use of psychoactive drugs.^17^ A study by Hildt et al.^8^ emphasized that there was a scarcity of real observed cognitive effects, given that the students reported that there was an increase in concentration and memory capacity after using such drugs. However, despite this positive subjective experience, few students reported that drug use had any real objective effect on academic outcomes. This raises the hypothesis that there might be a motivational effect associated with psychoactive drugs that is more important than the real effect on individual capacities.^8^ In some studies, many students on medications reported having high stress levels, especially before final exams or the due dates for handing in papers.^9^^,^^18^^,^^19^

In the present study, medical students showed higher prevalence of psychoactive drug use than dental students. In a study conducted by Webb et al.^13^ among medical students in the United States, it was suggested that 15% of the students were using stimulants during the academic course. It was found that 83% of the students were using stimulants specifically to improve academic performance.

Several authors have pointed out that the peculiar characteristics of medical schools may be contributing to the increased use of psychoactive substances among students. These characteristics include high course load, responsibility for patient healing, ethical issues, deaths of patients under student care and facilitated access to certain drugs that are restricted to healthcare professionals.^20^

In the present study, most of the students reported using this type of medication for the purpose of relaxation or stress relief (73.2%). Medical school has been described as a source of stress for students, who report especially the loss of personal freedom, excessive academic pressures and feelings of dehumanization.^21^ They also complain about the lack of leisure time and the strong competition among colleagues. These and other factors such as contact with sick patients predispose towards appearance of depressive conditions, anxious reactions, obsessive-compulsive neurosis and hypochondria, which lead students to use psychoactive drugs.^22^

Some studies have already proven the high prevalence of stress, anxiety and depression among medical students.^23^ According to the study by Mesquita et al.,^24^ the students themselves believe that the stress of medical school is a major factor for their use of drugs, particularly the stress relating to competitiveness, the intense course load, the abrupt transition from theoretical activities to practice and the medical residency test.

Medical students in the United States have been found to be more susceptible to developing symptoms of stress and dissatisfaction, and greater severity of signs of psychiatric illnesses.^25^^,^^26^ Medical students have been selected as the target population in a number of studies, which shows that there is higher concern regarding this specific group, given these individuals’ greater knowledge of pharmacology and greater access to these substances.^27^^,^^28^^,^^29^

In another study, in which medical students stood out because of their facilitated access to these substances, the euphoric effect associated with recreational use was emphasized as an explanation for non-medical consumption of psychoactive substances.^30^

Consumption of psychoactive drugs has produced social and health problems worldwide, especially because of the increasing prevalence of these drugs.^14^ Therefore, there is a need to implement interventions and increase student awareness about the potential effects produced by these substances. It is also crucial for this issue to be addressed in healthcare courses, because students can be considered to be an at-risk group regarding the use of psychostimulants and, in the future, they may be faced with this situation as professionals.^31^

According to Dal Pizzol et al.,^32^ the guidelines for public actions to prevent drug misuse should include educational campaigns targeted to the youth population with an emphasis on the most common psychoactive drugs. In addition, control over sales to minors and sales that are contrary to other restrictions established through health surveillance legislation should be reinforced.

One of the main areas of intervention will lie within improving the information and awareness of this student population regarding psychoactive drugs and their clinical and ethical effects, so as to prevent use and decrease the number of users.

Few scientific data on the effects of psychoactive substances or even on their long-term use have been published in the literature. Many of these studies have only indicated the growing trend in use among young people, without estimating further side effects, addictions or pathological conditions. Precisely because of the lack of data, the aim in this study was to ascertain the degree of use of psychoactive drugs among young students, in order to correlate this with deficiencies in their academic performance. Additionally, the aim was to observe whether the students were under medical care or were self-medicating, which is another matter of controversy within the academic environment. From analysis on the results, a high prevalence of psychoactive drug use was found, thus corroborating the scientific evidence.

The present study may have provided an important tool for further scientific studies focusing on the academic population of university courses with high course loads. From reflection on the increasing use of psychoactive drugs, it can be noted that this topic requires greater attention from the scientific community, to augment the sparse literature that currently exists and to obtain further data and reach more conclusions on this topic.

Therefore, it is necessary to implement interventions and increase the population’s awareness of the potential effects produced by psychoactive substances. Considering that the participants in the present study were healthcare students, it was expected that the consumption of stimulants would be lower, because such students are aware of the risks of these substances. However, what was most striking to us was the fact that most of these users had a medical prescription for the use of such drugs.

## CONCLUSION

From the results obtained, it was possible to conclude that:

- The use of psychoactive drugs among healthcare students at the higher education institution investigated was high, even though this use was via medical prescription.
- There was an association between the use of psychoactive drugs and some factors, with higher prevalence among women, medical students and subjects with lower-than-expected academic performance.
